# How Negative Experience Influences the Brain: A Comprehensive Review of the Neurobiological Underpinnings of Nocebo Hyperalgesia

**DOI:** 10.3389/fnins.2021.652552

**Published:** 2021-03-24

**Authors:** Mia A. Thomaidou, Kaya J. Peerdeman, Melissa I. Koppeschaar, Andrea W. M. Evers, Dieuwke S. Veldhuijzen

**Affiliations:** ^1^Health, Medical & Neuropsychology Unit, Leiden University, Leiden, Netherlands; ^2^Leiden Institute for Brain and Cognition, Leiden, Netherlands; ^3^Medical Delta Healthy Society, Leiden University, Technical University Delft, & Erasmus University Rotterdam, Netherlands; ^4^Department of Psychiatry, Leiden University Medical Centre, Leiden, Netherlands

**Keywords:** nocebo, hyperalgesia, learning, pain, neurobiology, neurophysiology, fMRI, EEG

## Abstract

This comprehensive review summarizes and interprets the neurobiological correlates of nocebo hyperalgesia in healthy humans. Nocebo hyperalgesia refers to increased pain sensitivity resulting from negative experiences and is thought to be an important variable influencing the experience of pain in healthy and patient populations. The young nocebo field has employed various methods to unravel the complex neurobiology of this phenomenon and has yielded diverse results. To comprehend and utilize current knowledge, an up-to-date, complete review of this literature is necessary. PubMed and PsychInfo databases were searched to identify studies examining nocebo hyperalgesia while utilizing neurobiological measures. The final selection included 22 articles. Electrophysiological findings pointed toward the involvement of cognitive-affective processes, e.g., modulation of alpha and gamma oscillatory activity and P2 component. Findings were not consistent on whether anxiety-related biochemicals such as cortisol plays a role in nocebo hyperalgesia but showed an involvement of the cyclooxygenase-prostaglandin pathway, endogenous opioids, and dopamine. Structural and functional neuroimaging findings demonstrated that nocebo hyperalgesia amplified pain signals in the spinal cord and brain regions involved in sensory and cognitive-affective processing including the prefrontal cortex, insula, amygdala, and hippocampus. These findings are an important step toward identifying the neurobiological mechanisms through which nocebo effects may exacerbate pain. Results from the studies reviewed are discussed in relation to cognitive-affective and physiological processes involved in nocebo and pain. One major limitation arising from this review is the inconsistency in methods and results in the nocebo field. Yet, while current findings are diverse and lack replication, methodological differences are able to inform our understanding of the results. We provide insights into the complexities and involvement of neurobiological processes in nocebo hyperalgesia and call for more consistency and replication studies. By summarizing and interpreting the challenging and complex neurobiological nocebo studies this review contributes, not only to our understanding of the mechanisms through which nocebo effects exacerbate pain, but also to our understanding of current shortcomings in this field of neurobiological research.

## Introduction

Negative thinking, such as having negative outcome expectations, can blunt the effect of active treatments, enhance the experience of aversive side-effects, and even produce deleterious effects in relation to recovery from symptoms such as pain, all leading to a phenomenon known as nocebo hyperalgesia (Colloca and Miller, 2011; Atlas and Wager, 2012; Colloca, 2014). Nocebo hyperalgesia refers to increased pain sensitivity and increased pain reports that mainly result from negative outcome expectations. Nocebo effects have been shown to be most relevant for alterations in pain tolerance or intensity and lead to higher pain reports when compared to baseline or control stimulations (Colloca et al., 2010; Albu and Meagher, 2016; Piedimonte et al., 2017). The neurobiological correlates of nocebo hyperalgesia are gaining research attention, but in lack of a comprehensive summary of findings, the neurobiology of these effects remains poorly understood. Gaining a better understanding of nocebo hyperalgesia on pain and its neural signature is an important step in the detection and prevention of these effects in patients, as well as the development of methods that may potentially counteract nocebo hyperalgesia.

**FIGURE 1:**
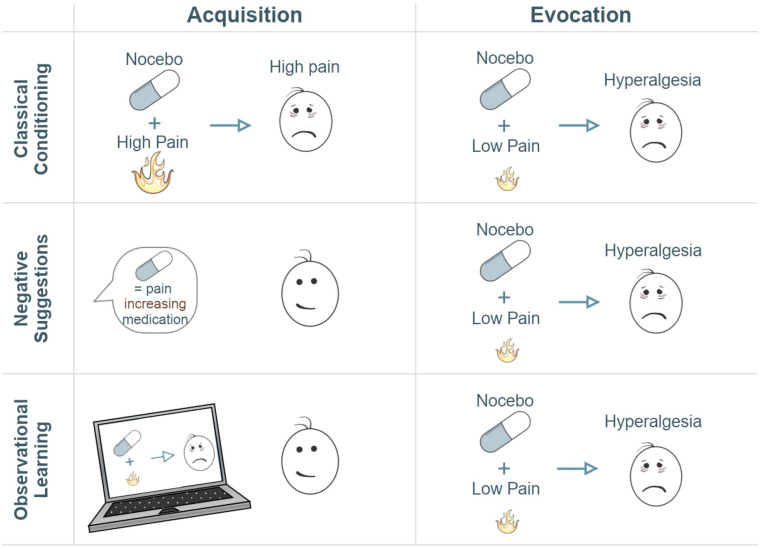
Illustration of three typical experimental paradigms for the induction of nocebo hyperalgesia. The acquisition column refers to the learning phase, whether conditioning-mediated, verbal, or observational learning. The evocation column refers to the evocation of the learned effect. Typically, an acquisition phase serves to induce negative expectations via conditioning, negative suggestions, observational learning, or any combination of these methods. Negative expectations are induced by combining an inert treatment with a surreptitious increase in pain stimulation (conditioning), by being told that a treatment will lead to increased pain sensitivity (negative suggestion), and/or by observing this negative treatment effect on someone else (observational learning). Subsequently, lower pain stimulations are administered in combination with the nocebo treatment in order to test whether nocebo hyperalgesia has been induced. A control group or condition where no nocebo is administered typically serves as a comparison to measure the magnitude of responses to the nocebo treatment. In this illustration, neutral faces express that there is no high-pain experience whereas sad faces represent the experience of high pain.

Studies examining the neural correlates of nocebo hyperalgesia utilize (combinations of) different learning processes to induce nocebo hyperalgesia experimentally in order to explore the various mechanisms by which pain circuitry and experienced pain can be modulated (Figure 1; Ellerbrock et al., 2015; Freeman et al., 2015; Ba̧bel et al., 2017): classical conditioning, instructional learning (i.e., through verbal suggestions), and social observational learning (Colloca et al., 2008; Bräscher et al., 2018). Classical conditioning forms and reinforces expectations through associative learning (Stockhorst, 2005). In conditioning models of nocebo hyperalgesia, an association is formed by repeated pairing between a high pain stimulus and an initially neutral stimulus (e.g., an inert treatment), that later becomes the nocebo stimulus. After repeated trials, an association is formed between the nocebo stimulus and the worsening of pain. Due to this negative expectation, the nocebo stimulus evokes increases in perceived pain, similar to the high pain intensity previously paired to the nocebo, even in the absence of high pain applications. Negative verbal suggestions can also alter pain expectations, through more explicit, instructional learning. Suggestions may induce negative expectations explicitly (i.e., explaining the potential negative effects of a treatment) and can also induce nocebo hyperalgesia for example by enhancing conditioning effects (Van Laarhoven et al., 2011; Bräscher et al., 2017). Social observational learning can moreover induce nocebo hyperalgesia, for example when one sees someone else experiencing increased pain after a treatment (Vögtle et al., 2013; Tu et al., 2019). These learning processes may result in nocebo responses that may play a detrimental role in shaping pain responses following a given event, stimulus, or treatment (Colloca and Miller, 2011; Manaï et al., 2019).

Over the past two decades, neuroimaging and pharmacological studies have begun to address the neurobiological underpinnings of nocebo experiences. Electrophysiological and neuroimaging methods such as electroencephalography (EEG) and magnetic resonance imaging (MRI) have provided valuable insights into the specific functional brain processes and underlying brain structures that are involved in nocebo hyperalgesia (Kong et al., 2008; Scott et al., 2008; Schmid et al., 2015; Albu and Meagher, 2016; Thomaidou et al., 2021a). Moreover, the neurochemical systems underlying nocebo hyperalgesia have been explored via pharmacological administrations, blood or salivary measurements, or via imaging techniques such as Positron Emission Tomography (PET; Benedetti et al., 2006, 2014; Scott et al., 2008). Inconsistencies and gaps in the literature, however, render nocebo hyperalgesia a phenomenon that is still poorly understood. The nocebo literature is characterized by very diverse methods. For this reason, a comprehensive and detailed account of studies that examined neurobiological correlates of nocebo hyperalgesia may significantly aid in a better understanding of this phenomenon and can provide suggestions for improvements in the consistency of selected methodologies and reporting of results.

Through this review, we intend to provide a detailed overview of current neurobiological nocebo studies on pain and their findings. While a systematic review on this topic was not preferable due to the scarcity and diversity of neurobiological nocebo studies, a comprehensive and detailed account of these studies could be very valuable. First, in Supplementary Table 1, we briefly list the different experimental models used to induce nocebo hyperalgesia across the included studies (Supplementary Table 1), especially when paradigms deviated from typical nocebo induction methods. We then provide a comprehensive overview of electrophysiological, neurochemical, and structural and functional correlates of nocebo hyperalgesia in healthy humans. These findings are discussed in relation to the sensory and cognitive processes involved, thereby providing a clear and comprehensive overview of the multitude of brain correlates involved in nocebo hyperalgesia. Finally, recommendations are provided to use more consistent methodologies and reporting of results and for replication studies.

## Selection of Studies

**FIGURE 2:**
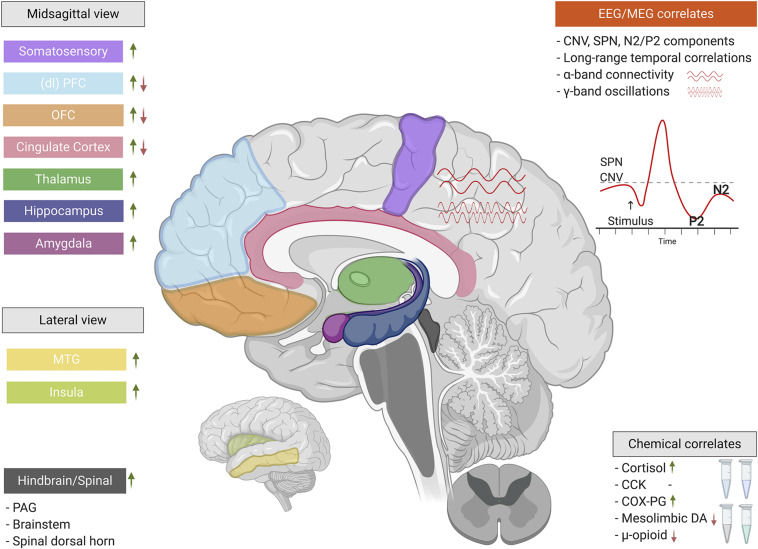
The neurobiological correlates of nocebo hyperalgesia. When classical conditioning and/or negative suggestions are used to experimentally induce nocebo hyperalgesia, a complex interplay of electrophysiology, neurochemistry, and central nervous system functionality come into play. These neurobiological factors involve a wide array of functions ranging from basic nociceptive to cognitive-affective. Green upward arrows indicate increases/activations of particular regions, components, or chemicals, while red downward arrows indicate decreases/deactivations (*CCK’s role cannot be simplified by a red/green arrow). EEG, electroencephalography; MEG, magnetoencephalography; SPN, stimulus-preceding negativity; CNV, contingent negative variation; (dl)PFC, (dorsolateral) prefrontal cortex; OFC, orbitofrontal cortex; MTG, middle temporal gyrus; PAG, periaqueductal gray; CCK, cholecystokinin; COX-PG, cyclooxygenase-prostaglandin pathway; DA, dopamine. This figure was created using BioRender.com.

A search strategy was used to identify studies on nocebo hyperalgesia on PubMed and PsychInfo, published up to July 2020, using detailed key terms related to nocebo hyperalgesia, nocebo conditioning, and neurobiological methods (Supplementary Material). The abstracts of 1,761 articles were screened for inclusion by the first author. When there was doubt about inclusion of a study, a decision was made in consultation with the last author. This review focuses only on nocebo hyperalgesia induced in healthy humans, in order to summarize and compare findings that are not influenced by underlying pain or psychological conditions and as such most clearly present the underlying mechanisms of nocebo hyperalgesic effects. Exclusion criteria were: (1) not using an experimental learning paradigm for the induction of nocebo hyperalgesia, (2) not utilizing a healthy human sample, (3) not utilizing at least one neurobiological measure, such as brain imaging or a pharmacological manipulation, and (4) not inducing significant nocebo responses (as neurobiological responses in relation to nocebo hyperalgesia can only be studied following a successful nocebo manipulation). The articles that fit the inclusion criteria were considered relevant for understanding the neurobiological underpinnings of nocebo hyperalgesia and are described in detail in this narrative review. The final selection included 22 articles (Supplementary Table 1), of which six articles reported electrophysiological measures, three focused on chemical correlates, one reported structural brain correlates, and 12 reported functional brain correlates using functional magnetic resonance imaging (fMRI) results. Because we selectively focused on the neurobiology of nocebo hyperalgesia, we report only the nocebo arms of studies (for example, we do not report placebo manipulations). Figure 2 provides an illustration of all major findings of the studies reviewed here.

## Electrophysiological Correlates of Nocebo Hyperalgesia

Electroencephalography and magnetoencephalography (MEG) are non-invasive imaging techniques that either directly or indirectly, respectively, measure electrical activity in the brain through electrodes placed on the scalp (Niedermeyer et al., 2010; Proudfoot et al., 2014). Neuronal oscillations in the classical frequency bands as well as neuronal (de)activations in response to a specific stimulation have now been consistently related to sensory, cognitive, and affective processes (Klimesch, 1999; Bell and Diaz, 2012; Pinheiro et al., 2016; Banich and Floresco, 2019). EEG and MEG are thus valuable techniques for unraveling the neurophysiology underlying nocebo hyperalgesia. Six studies to date have used these methods to examine nocebo-related resting-state or event-specific alterations. Of these six studies, one used negative suggestion alone (Albu and Meagher, 2016) and five used conditioning methods to induce nocebo hyperalgesia. Conditioning was used with (Thomaidou et al., 2021a) or without (Hird et al., 2018) negative suggestions, while two studies examined conditioning in separate groups either with or without negative suggestions (Pazzaglia et al., 2016; Piedimonte et al., 2017) and in one (MEG) study conditioning was combined with observational learning (Tu et al., 2019).

Albu and Meagher (2016) investigated alterations in EEG activity in a study using negative verbal suggestions regarding inert nocebo and control creams. The nocebo manipulation resulted in a significant increase in thermal pain ratings. Moreover, a significant increase in low alpha EEG power (8–10 Hz) was found in the nocebo group relative to the control group when comparing a 5-min EEG recording during noxious heat stimulation pre- to post-acquisition of the nocebo effect. The exact topography of this finding was not specified. This change in low alpha activity, however, correlated to an increase in pain catastrophizing and not to an increase in pain ratings. Pain catastrophizing in this study was measured via the Pain Catastrophizing Scale (Sullivan et al., 1995) that assesses catastrophizing thoughts related to pain, or pain-related worrying (Crombez et al., 2020). The authors suggested that the increase in low alpha power reflects a negative cognitive-affective state in relation to pain, in a process parallel to, or potentially involved in, nocebo hyperalgesia.

Thomaidou et al. (2021a) studied electrophysiological processes underlying nocebo hyperalgesia, aiming to identify EEG biomarkers of nocebo-augmented pain. Nocebo effects on thermal pain were induced through conditioning and negative suggestions regarding the pain increasing effects of an inert gel. Nocebo hyperalgesia led to widespread pre- to post-acquisition increases in resting-state long-range temporal correlations of brain oscillations, which were negatively associated with nocebo magnitudes. Individuals with strong long-range temporal correlations of brain oscillations during pre-acquisition rest showed larger nocebo responses than those with weak long-range temporal correlations. Moreover, increases in alpha and decreases in beta and gamma oscillations were found during nocebo-augmented pain in the evocation phase. This study highlighted the role of increased cognitive processing of pain at the electrophysiological level, under nocebo-hyperalgesic conditions.

Pazzaglia et al. (2016) used laser pulses to measure laser-evoked potentials (LEPs), aiming to investigate potentials related to cognitive control, such as the N2 and P2 components. In two groups, either negative suggestions in combination with conditioning or negative suggestions alone about a nocebo cream were used to induce nocebo effects. A neutral cream was used as the control stimulus. Post-treatment pain ratings were compared to baseline and between the cream-treated hand and the untreated hand. The authors demonstrated reduced habituation to pain as a result of the nocebo manipulations. Diminished N2/P2 LEP amplitudes in central scalp regions paralleled the diminished habituation.

Piedimonte et al. (2017) aimed to differentiate between specific sensory-anticipatory and motor components of electrically induced nocebo hyperalgesia. To this end, the authors examined contingent negative variation (CNV) amplitudes. Early CNV is considered an event-related potential related to the anticipation of an upcoming event, while late CNV is considered to be related to motor preparation for an event (Brunia and van Boxtel, 2001; Chiu et al., 2004; Nagai et al., 2004). Early CNV component amplitudes showed significantly higher early negativity in nocebo trials, as demonstrated by a comparison between placebo cues (signaling a decrease in pain) and nocebo cues (signaling an increase in pain) during acquisition and evocation and in frontal, central, and parietal brain regions. Differences in late negativity were not found during nocebo evocation, suggesting that the motor reaction to pain may not be affected by nocebo hyperalgesia. The authors conclude that based on their results, expectation of hyperalgesia may affect the early, sensory component of pain, thereby producing a modulation of pain perception. The expectation of hyperalgesia under nocebo conditions seems to be related, based on this study, to the perception of increased pain, via an “early” cognitive mechanism that anticipates or prepares for a highly painful stimulus.

Hird et al. (2018) used both electric and laser-evoked pain which allowed testing for time-sensitive EEG components, while also contrasting the effects of different types of pain on brain signals. Electric-evoked potentials (EEPs) and LEPs were recorded throughout the experiment while participants underwent the nocebo manipulation. An effect of the nocebo manipulation on pain ratings was found, suggesting that, compared to the placebo cue (signaling a 75% likelihood of low pain), the nocebo cue (signaling a 75% likelihood of high pain) increased ratings for stimuli of moderate intensity, in response to both laser and electric stimulation. Hird et al. (2018) investigated the stimulus-preceding negativity (SPN) component, which is thought to be a slow-wave EEG correlate of imminent pain anticipation. The SPN at centroparietal electrodes was found to differentiate pain intensity expectation (i.e., anticipation of high pain intensity versus low pain intensity) with nocebo trials linked to more negative amplitudes. This was found in response to laser-evoked pain, but not to electric pain stimuli, indicating morphological differences in brain activations between the two stimulation types. The topographical findings were connected to previous studies indicating sources in the anterior insula and cingulate cortex (Brown et al., 2008).

Tu et al. (2019) aimed to study distinct learning processes of nocebo effects on thermal pain, using MEG. Classical conditioning and observational learning were compared and both conscious and unconscious visual cues were used. In the classical conditioning phase, participants were asked to learn the associations between neutral faces presented on a screen and the experience of low and high pain. In the observational learning phase, a different pair of faces were accompanied by observing a model experiencing and rating low and high pain. Resting-state MEG data were recorded twice for each subject, before and after conditioning. All nocebo manipulations significantly induced nocebo hyperalgesia and significant changes in brain connectivity were demonstrated after conditioning across all frequency bands. A decrease in alpha band connectivity between the left rostral anterior cingulate cortex (rACC) and left middle temporal gyrus (MTG) was the most consistent predictor of the magnitude of induced nocebo effects across all manipulations. The authors discuss their finding in relation to earlier imaging research linking the rACC, a primary center for sensory-discriminative processing (Tinnermann et al., 2017), with the nocebo effect.

In sum, with electrophysiological studies in the nocebo field being limited, the few studies that have explored the electrophysiological correlates of nocebo hyperalgesia have focused on different aspects. None of the studies described in this review used similar behavioral or imaging analysis methods therefore challenging the comparison of results. What seems to be a recurrent pattern in these studies is the involvement of brain components in nocebo hyperalgesia that have previously been implicated in cognitive and affective processes. This is demonstrated by activations in (low) alpha band activity, the early CNV component, and SPN component, reductions in the N2/P2 LEP component and alpha connectivity and with source regions for these diverse results identified in the anterior insula, cingulate gyrus, and MTG (Albu and Meagher, 2016; Pazzaglia et al., 2016; Piedimonte et al., 2017; Tu et al., 2019). Moreover, learning has been implicated at an electrophysiological level, as shown by alterations in gamma band activity under nocebo hyperalgesic conditions as well as the involvement of long-range temporal correlations (Thomaidou et al., 2021a).

## Neurochemical and Biochemical Correlates of Nocebo Hyperalgesia

Neurochemicals play a key role in nociception and in the cognitive and affective processes that modulate pain perception (Osterweis et al., 1987). In nocebo hyperalgesia, where cognitive and emotional factors such as learning and anxiety have been shown to play a role (Colloca and Benedetti, 2007; Egorova et al., 2015; Colagiuri and Quinn, 2018), related neurochemicals may be involved. Next to neurochemicals, other biochemicals such as enzymes have been shown to modulate pain transmission (Basbaum et al., 2009) and may also be relevant in nocebo hyperalgesia. Three studies examined chemical processes involved in nocebo hyperalgesia. All three studies used negative suggestions to induce nocebo hyperalgesia (Benedetti et al., 2006, 2014; Scott et al., 2008).

Benedetti et al. (2006) studied cortisol and the hypothalamic–pituitary–adrenal (HPA) axis by using a neuropharmacological approach to examine neurochemical correlates of anxiety, a state that is related to fear and may thus, similarly to fear (Thomaidou et al., 2021b), also be linked to nocebo hyperalgesia (Colloca and Benedetti, 2007). Participants were subdivided into 4 groups and underwent ischemic pain inductions. One group received a sham hyperalgesic pill and intravenous proglumide, a non-selective antagonist of cholecystokinin (CCK) type-A/B receptors (Bunney et al., 1985). A second group received a sham hyperalgesic pill and intravenous diazepam, a benzodiazepine and potent anxiolytic agent that increases the effect of the inhibitory neurotransmitter gamma-aminobutyric acid (Riss et al., 2008). A nocebo control group received a sham hyperalgesic pill and an inert saline solution, while the other control group was only administered a saline solution and no nocebo manipulation. In the nocebo control group, significant nocebo hyperalgesia and HPA axis hyperactivity were observed, as shown by increased adrenocorticotropic hormone (ACTH) and cortisol plasma concentrations. Proglumide administration only blocked nocebo hyperalgesia reports but not the nocebo-induced hyperactivity of the HPA axis, while diazepam blocked both nocebo hyperalgesia and nocebo-induced HPA axis hyperactivity. Based on these results, the authors suggested that the CCK antagonist proglumide may act on anxiety-induced hyperalgesia, as proglumide affected pain but not the HPA axis. In relation to their findings that highlight two different anxiety pathways for HPA hyperactivity and hyperalgesia, they discuss that hyperalgesia may occur when anticipatory anxiety is about the pain itself (Benedetti et al., 1997).

In a later study, Benedetti et al. (2014) studied nocebo effects on hypoxia-induced headache, a symptom experienced at high altitudes due to the altered synthesis of eicosanoid signaling molecules, such as prostaglandin (PG) and thromboxane A_2_ (TXA_2_), through the cyclooxygenase (COX) enzyme at height (Richalet et al., 1991). Blockade of PG synthesis with aspirin can prevent high altitude headache (Burtscher et al., 1998). In a social nocebo manipulation, prior to a mountaineering trip, researchers provided negative suggestions to only one participant, who communicated the suggestions to 35 of his peers. Of the participants not reached by the nocebo suggestions, 38 were allocated to a control group. Participants visited a research location at an altitude of 3,500 m, where headache sufferers were further subdivided into groups that received aspirin (25 mg/kg), placebo, or no treatment. Salivary PG, TXA_2_, and cortisol were measured at sea level and at 3,500 m. Additionally, identical nocebo and control groups extracted from a separate participant sample went up to only 1,500 m, where no hypoxia is supposed to take place, and underwent the same procedures. At 3,500 m, headache occurrence and intensity were significantly higher in the nocebo group relative to the control group. Larger increases in PG and TXA_2_ were found in the nocebo group relative to the control group. Cortisol increase was found only in the nocebo group. Aspirin relieved the headache and blocked PG and TXA_2_ increases in headache sufferers of the nocebo and control groups, while placebo administration had these effects only in the nocebo group. At 1,500 m there were no significant nocebo effects or increases in PG and TXA_2_. The authors concluded that socially induced nocebo effects affected the biochemical pathway related to PG synthesis; however, negative expectations were insufficient in initiating pain and PG synthesis in the absence of hypoxia. This study indicates that nocebo hyperalgesia can affect peripheral biochemical pain mechanisms.

In a PET study, Scott et al. (2008) examined the contribution of the endogenous opioid and dopaminergic (DA) systems in the induction of placebo hypoalgesia. Participants underwent intramuscular pain inductions and four PET scans were obtained, two with and two without placebo administration. While this study aimed to induce placebo effects, five participants showed significant increases in pain reports during placebo administration who can be considered nocebo responders. The researchers found significant changes in μ-opioid and DA (D_2_/D_3_ receptor) neurotransmission between high placebo and nocebo responders. Compared to placebo responders, nocebo responders demonstrated a deactivation of μ-opioid and DA neurotransmission in specific brain regions: the right nucleus accumbens and left ventral putamen. For μ-opioids these regions additionally included the nucleus accumbens, subgenual ACC, orbitofrontal cortex (OFC), anterior insula, periaqueductal gray (PAG), mediodorsal area of the thalamus, and amygdala. Notably, the regions and neurotransmitter systems involved in placebo and nocebo effects overlapped.

Collectively, these studies have contributed to an early understanding of biochemical variables that may be implicated in nocebo hyperalgesia. The studies reviewed here, however, employed different pain induction methods and generally focused on discrete chemicals. Of these chemicals, ACTH, PG, opioids, and dopamine seem to be involved in nocebo hyperalgesia. Moreover, the stress hormone cortisol has been a recurrent focus of the few neurochemical nocebo studies and seems to play a role in nocebo hyperalgesia.

## Functional and Structural Correlates of Nocebo Hyperalgesia

### Transcranial Direct Current stimulation

Transcranial Direct Current Stimulation (tDCS) is a non-invasive neuromodulatory technique which delivers low electrical currents via scalp electrodes that can increase or decrease neuronal excitability (Kuo and Nitsche, 2015; Hill et al., 2016). When positive stimulation (anodal tDCS) is delivered, neuronal excitability is increased (Kuo and Nitsche, 2015). When negative stimulation (cathodal tDCS) is delivered, there is a decrease in neuronal excitability (Kuo and Nitsche, 2015). Among other uses, tDCS can help investigate whether a brain region of interest is implicated in a specific process, such as the acquisition or the evocation of nocebo hyperalgesic responses. One study looked at the influence of tDCS on nocebo hyperalgesia using a conditioning paradigm (Egorova et al., 2015).

Egorova et al. (2015) aimed to modulate nocebo effects by altering the excitability of the right dorsolateral prefrontal cortex (rDLPFC) using tDCS. Thirty participants were randomized into two tDCS groups that received either anodal or cathodal rDLPFC stimulation. Bipolar tDCS was administered to the rDLPFC for 20 min during rest, after nocebo conditioning and before nocebo evocation. During the conditioning phase, geometric fractal shapes were used as visual cues, paired to high and low pain stimulations. A significant nocebo effect was induced only in the anodal tDCS group, indicating that conditioned nocebo effects can be elicited after anodal but not cathodal rDLPFC stimulation. This study was not able to unequivocally determine whether tDCS led to enhancement of nocebo hyperalgesia in the anodal condition and/or whether it caused a reduction in the cathodal condition. However, the authors speculated, based on earlier literature (Jensen et al., 2012), that cathodal tDCS presumably reduced the effects of conditioning.

### Functional Imaging

Functional magnetic resonance imaging is a technique that can map brain function by detecting blood-oxygen-level dependent (BOLD) changes and thereby indirectly measuring brain activity (Logothetis et al., 2001; Huettel et al., 2009). The measurement of BOLD activity in the brain has helped researchers explore functional brain correlates, from sensory perception to higher cognitive functions such as the formation of expectations (Simpson et al., 2001; Phan et al., 2002; Atlas and Wager, 2012; Wager and Atlas, 2015). When comparing the frequently used brain imaging techniques EEG and fMRI, fMRI provides higher spatial resolution whereas EEG provides greater temporal resolution. MRI also enables high resolution structural brain imaging. In the 12 studies reviewed below, fMRI has shed light onto some of the functional and anatomical similarities and differences between nocebo hyperalgesia and related effects such as placebo analgesia. These studies used various nocebo induction methods: 3 used conditioning (Keltner, 2006; Kong et al., 2013; Jensen et al., 2015), 4 used negative suggestions (Rodriguez-Raecke et al., 2010; Schmid et al., 2013, 2015; Ellerbrock et al., 2015), and 5 used a combination of conditioning and negative suggestions (Kong et al., 2008; Geuter and Buchel, 2013; Freeman et al., 2015; Tinnermann et al., 2017; Egorova et al., 2020). Studies that described nocebo effects in somatic pain, visceral pain, or spinal pain are summarized separately because of the methodological differences in the study designs and their proposed underlying mechanisms.

Eight studies examined brain activation in response to nocebo hyperalgesia for somatic pain using fMRI. In a study by Keltner (2006), participants were conditioned to associate two distinct visual cues (red or blue) with thermal pain stimuli of high and low intensity, respectively. The imaging data of the nocebo evocation phase revealed that during high intensity pain stimulation, high and low pain expectations produced differential brain activations. The caudal ACC, the cerebellum, and the dorsolateral pontomesencephalic region had increased BOLD activation when expectations were for higher pain intensity. During low intensity pain stimulation, the two expectation levels did, however, not yield significant differences in BOLD activations. The authors stated that this difference indicated that negative expectations in combination with high pain stimulation elicited a sum of neural activity that enhanced activation of afferent pain circuitry. In the context of descending pain modulation, expectation and pain intensity may have acted in an additive manner on afferent pathways when these were activated by high pain stimulation.

Kong et al. (2008) informed their participants that a (sham) acupuncture treatment on the arm may increase their pain sensitivity while also conditioning them with surreptitiously increased thermal pain stimulations. During the fMRI session, participants underwent the nocebo manipulation again and then received all pain stimulations at moderate intensity. After administering the inert treatment, pain intensity ratings significantly increased for the nocebo sites compared to control sites on the arm. Pre- and post-treatment differences in brain activations revealed significant increases in activations during nocebo, as compared to control trials, in the dorsal ACC, insula, superior temporal gyrus (STG), left frontal and parietal operculum, medial frontal gyrus, OFC, superior parietal lobule, hippocampus, right putamen, lateral PFC, and MTG. Furthermore, significant positive correlations were observed between nocebo magnitudes and activations in the bilateral insula and left primary motor cortex. Significant negative correlations were observed between nocebo magnitudes and activations in the dlPFC and left OFC. Activation differences in these brain regions suggest that nocebo hyperalgesia predominantly engaged the affective-cognitive pain circuit.

In a later study, Kong et al. (2013) studied pain expectations but now without employing a sham treatment. In this fMRI study, visual cues were associated with high thermal pain stimulations. When predictive cues were paired with moderate pain intensity, nocebo hyperalgesic responses were reported. fMRI data from the evocation of nocebo hyperalgesia did not yield major findings related to the experience of pain following the high-pain visual cue. However, the researchers analyzed pretest resting state fMRI data and found that functional connectivity between frontoparietal regions and the rACC and medial PFC was positively associated with nocebo responses. These data suggested that a frontoparietal network controlling top-down regulation of pain and other incoming information (Miller, 2000) may also be involved in the processing of pain under nocebo hyperalgesic conditions.

Rodriguez-Raecke et al. (2010) implemented a novel nocebo paradigm by carrying out a longitudinal nocebo experiment, focusing on (lack of) reduced habituation. Participants in one group were told that over eight testing sessions they would become more sensitive to repeated thermal pain stimulations. The control group was not given any information and thus was expected to exhibit typical habituation to thermal pain. Indeed, while the control group showed habituation, the nocebo group did not report significantly lower pain levels over time, indicating that the acquisition of a nocebo response was successful. fMRI data were collected on day 1 and day 8 of the experiment. These data showed predominantly significantly increased activation of the operculum in the nocebo, as compared to the control group. Differences in activation of the operculum indicated a potential involvement of early nociceptive processing in nocebo hyperalgesia (Greenspan et al., 1999).

In another longitudinal experiment, Ellerbrock et al. (2015) tested participants for 21 consecutive days, with fMRI scanning taking place on days 1, 8, 14, and 21. The nocebo manipulation consisted of a negative suggestion aiming to reduce habituation to pain. Participants in this nocebo group were told that in an earlier study, repetitive thermal pain stimuli were increasingly painful over days. Participants in the control group were not given suggestions. Negative suggestions resulted in activation of the operculum, such that it was increasingly activated over time in the nocebo group. Moreover, the nocebo induction largely inhibited activation in the PAG for the nocebo group relative to the control group. Across both groups, the operculum exhibited a gradual decrease in connectivity with the basal ganglia and a gradual increase in connectivity with the STG. However, no differences in connectivity were identified between the nocebo and control groups. The authors suggested that nocebo suggestions may modulate the contribution of the operculum on a pain-transmitting process that involves basal ganglia–thalamocortical loops. Importantly, the nocebo-mediated inhibition of PAG activation suggested that nocebo effects may impede descending pain modulation.

Freeman et al. (2015) studied negative suggestions regarding a pain-increasing effect of an inert cream labeled “Capsaicin” which was delivered to participants over three experimental sessions. In the first session, pain calibrations were conducted. In the second and third session, baseline pain stimuli were administered at a moderate intensity and short conditioning procedures took place. In the final part of the third session, the evocation phase took place inside the MR scanner. Negative suggestions significantly increased subjective pain ratings. The fMRI results showed that the expectation of increased pain induced significant BOLD activations in the insula, OFC, and PAG. While an involvement of the PAG in nocebo hyperalgesia has been found in previous research (Ellerbrock et al., 2015), it is notable that unlike in Ellerbrock et al. (2015), in this study PAG activation was increased in response to the nocebo manipulation.

Jensen et al. (2015) aimed to investigate the neural correlates of specifically non-conscious conditioned nocebo hyperalgesia. Participants were told to focus on images presented on a screen that would accompany pain stimulations and to rate their pain following each stimulus. They were then conditioned by use of images that depicted neutral male facial expressions, presented supraliminally. During the subsequent evocation phase, supraliminal and subliminal presentations of the conditioned faces were accompanied by moderate pain stimulations. Both conscious and non-conscious exposure to the conditioned cues led to significant nocebo responses. Nocebo hyperalgesia, irrespective of exposure type, revealed increased activation in several regions involved in nociceptive processing, such as the ACC, insula, thalamus, and brainstem. As compared to conscious nocebo, non-conscious nocebo led to increased activation of the thalamus, amygdala, and hippocampus. Involvement of these subcortical structures may reflect processing and encoding of a perceived threat (Phelps, 2004), given the aversive nature of pain.

In a recent study, Egorova et al. (2020), aimed to investigate the effects of administering an inert cream while providing neutral information regarding its effects on pain. This neutral condition was compared to a nocebo, in which negative suggestions were provided about the effects of a second cream. A conditioning paradigm was used in which increased pain was administered in the nocebo as compared to the neutral skin patches. The evocation phase took place inside the MRI scanner. While this study focused on how participants responded to pain following the administration of the neutral cream, connectivity analyses were conducted for the nocebo condition as well. These results showed increased connectivity between the left amygdala and the striatum and this increase was correlated with the magnitude of nocebo responses. With a particular focus on the amygdala, this study highlighted an involvement of the amygdala in modulating or reflecting the magnitude of nocebo responses.

Taken together, fMRI studies that explored brain correlates of nocebo effects on somatic pain have provided some consistent findings. As expected, sensory-discriminative and descending processing has been implicated in the presentation of nocebo hyperalgesic responses, with brain areas such as the ACC, operculum, PAG, and the PFC being consistently involved in nocebo hyperalgesia (Geuter and Buchel, 2013; Ellerbrock et al., 2015; Freeman et al., 2015). Studies consistently show that nocebo responses also implicate other cognitive as well as affective processes, as evident by the involvement of areas such as the OFC and DLPFC, ACC, insula, amygdala, and hippocampus (Kong et al., 2008; Scott et al., 2008; Jensen et al., 2015). Interestingly, studies that only employed conditioning but did not use negative suggestions to induce negative treatment expectations, did not observe an involvement of brain areas responsible for affective processes such as fear (Keltner, 2006; Kong et al., 2013).

### Functional Imaging in Visceral Models

Two studies investigated negative treatment expectations in an experimental model of visceral pain in which a pressure-controlled barostat system was used to inflated rectal balloons to an individualized designated pressure. Two studies used verbal suggestions and one used conditioning methods alone to induce nocebo effects. Schmid et al. (2013) told participants that they would experience increased pain as a result of receiving an opioid antagonist in one scanning session and saline solution in a control session. In reality, only saline was administered intravenously. Participants reported significantly higher pain levels during the expectation of a hyperalgesic treatment, as compared to the control sessions. The fMRI analyses indicated significantly increased pain-induced activation within the somatosensory cortex under nocebo conditions. Moreover, negative expectations in the nocebo group led to increased insula activation compared to neutral expectations.

In an fMRI study by this research group, Schmid et al. (2015) informed participants in a nocebo group that increased pain would occur over time due to sensitization, in response to repeated rectal distensions, while a control group did not receive any negative suggestions. In reality, previous work has revealed no evidence of sensitization (Elsenbruch et al., 2012). The nocebo group reported higher pain levels in the evocation phase as compared to the first session, indicating nocebo sensitization. When only nocebo responders (*n* = 14) were contrasted to the control group, greater activations were found in the amygdala and secondary somatosensory cortex during pain anticipation. During the pain inductions, nocebo responders demonstrated significantly enhanced hyperactivation of the amygdala, thalamus, and insula. As a function of negative expectations, the insular cortex showed increased connectivity with the midcingulate cortex (MCC) extending to the posterior cingulate cortex (PCC) during pain stimulations. Schmid et al. (2015) stated that their findings highlighted an involvement of the MCC in visceral nocebo effects.

In sum, visceral and somatic experimental models of nocebo hyperalgesia show a consistent involvement of cognitive-affective brain regions such as the hippocampus and amygdala. A consistent finding that seems to be prominent in visceral pain studies but also in somatic pain studies although somewhat less consistently, is the involvement of the insula in nocebo hyperalgesia. The insula is thought to be crucial for neural functions such as sensory integration and pain-related decision making (Craig, 2003; Veldhuijzen et al., 2010; Wiech et al., 2010; Linnman et al., 2011). The insula may thus constitute a primary brain region for the cognitive modulation of visceral and somatic pain (Auvray et al., 2010). Visceral pain studies have also found a role of the MCC in nocebo hyperalgesia, which was not observed in somatic pain studies. The MCC has been implicated in pain-related processes, including cognitive modulation and fear responses related to pain (Vogt, 2016), central sensitization to visceral pain and pain modulation in patients with chronic abdominal pain (Pereira et al., 2010; Elsenbruch, 2011; Hamaguchi et al., 2013; Vogt, 2013; Misra and Coombes, 2015). These findings suggest that nocebo effects on visceral pain show similarities to other types of pain. At the same time, these studies highlight a distinct implication of structures such as the MCC in visceral pain.

### Spinal Imaging

Functional magnetic resonance imaging has been used to image the function of the entire central nervous system. Because of specialized requirements for spinal MR images, however (Moffitt et al., 2005; Kornelsen and Mackey, 2007), neurobiological nocebo research has predominantly focused on brain mechanisms. Relatively recently, the focus has expanded to the spinal cord, which plays a key role, not only in the afferent transmission of pain signals, but also in the descending modulation of pain (Bingel and Tracey, 2008). In nocebo effects, hyperalgesia may be attributed to sensory and cognitive-emotional brain processes such as those described earlier. However, Benedetti et al. (2014) also showed that peripheral biochemical mechanisms may also play a role in nocebo effects. Whether there is an additional early or late source of increased pain perception in the spinal cord, is a question of high relevance and importance. Two studies examined spinal fMRI for nocebo hyperalgesia both induced by conditioning combined with verbal suggestions methods.

Geuter and Buchel (2013) investigated thermal conditioning combined with the suggestion that a (sham) capsaicin cream would enhance their perceived pain, while a control cream would have no effect on pain. Significant nocebo effects were reported. The fMRI results revealed that the nocebo manipulation led to increased BOLD signal in the ipsilateral dorsal horn of the spinal cord. Interestingly, the location of the nocebo-enhanced pain signal largely overlapped with the main effect of pain during heat stimulation. Moreover, response time to painful stimulation differed, with the signal increasing earlier when the nocebo treatment was applied compared to control. Overall, the findings demonstrate nocebo-induced increases in spinal pain signals, indicating that an early pain-facilitating mechanism takes place at the spinal level.

Tinnermann et al. (2017) integrated spinal and brain imaging, aiming to unravel whether nocebo hyperalgesia is mediated through cortico-subcortico-spinal network interactions, similarly to other forms of cognitive pain modulation. In this study, the impact of perceived value of a nocebo treatment on the magnitude of nocebo responses was also explored. Participants were allocated to one of two groups and received negative suggestions regarding the hyperalgesic effect of inert creams, one labeled as expensive and one as cheap. An additional neutral cream was used as a control. Nocebo effects were successfully induced and participants who received the sham expensive cream reported a significantly higher nocebo effect than participants who received the sham cheap cream. Regions that displayed neural representations of nocebo hyperalgesia irrespective of medication value were identified in the spinal cord at the height of spinal segment C6, slightly more caudal and medial than the pain cluster. This location was almost identical to the results of Geuter and Buchel (2013). Furthermore, compared to the cheap cream group, the expensive cream group had greater activation differences between nocebo and control trials in prefrontal areas, the right amygdala, and the PAG. Moreover, the level of rACC deactivation predicted the strength of reported nocebo hyperalgesia and the spinal cord and rACC revealed coupling with the PAG that correlated with the nocebo magnitude.

Taken together, these initial spinal imaging studies on nocebo hyperalgesia showed that both ascending and descending pain modulation at the spinal cord level may be involved in the presentation of nocebo effects. Modulation of the rACC-PAG-spinal axis could represent a mechanism through which the descending pain pathway interacts with higher-cognitive information, such as learned information or negative suggestions, to modulate pain processing. With the pain signal being amplified already at the spinal level, however, interesting questions may be raised regarding the contributing role of the spinal cord in pain amplification under nocebo conditions. While the observed amplification of spinal pain signals suggests a key role for spinal modulatory processes in nocebo hyperalgesia, further modulations at later, cortical areas remain important.

## Discussion

This review provides an overview of the neurobiological correlates of experimentally induced nocebo hyperalgesia. fMRI findings showed that activity might be amplified already in the spinal cord and further modulated by higher cognitive representations, such as cognitive and affective processes. Electrophysiological findings, though limited, also pointed toward involvement of cognitive-affective processes. Neurochemical findings were not consistent on whether cortisol may play a role in nocebo hyperalgesia, but pointed toward an involvement of specific endogenous neurotransmitter systems. Due to the multifaceted nature of nocebo hyperalgesia as a learned effect, physiological components remain difficult to disentangle from other variables, such as cognitive mechanisms related to sensory perception. Central issues arising from the compilation of neurobiological findings from the nocebo literature are the widespread inconsistency in methods used and results yielded, albeit this being understandable given the youth of the nocebo field. This diversity in methods and reporting of findings seriously challenges the interpretation of these findings. In discussing the results of this review, we have attempted to broadly categorize findings into neurobiological processes. It should be noted that this compartmentalization adds clarity to the interpretation of the results, the boundaries between these categories are blurred and categories largely overlap.

### Sensory Discrimination

Sensory discrimination allows for the processing of details both within the sensory input and between distinct types of sensations. It is unsurprising that this broad, primary type of pain processing is involved in nocebo hyperalgesia. Yet, findings that show increased involvement of sensory-discriminatory processes linked to nocebo, as compared to control pain, are very valuable. These findings reveal that typical perception of increased pain stimulation may involve very similar pain mechanisms as aggravated pain under hyperalgesic conditions (i.e., in the absence of increased pain stimulation). Electrophysiological findings showed the important involvement of sensory discrimination. Alpha activity has long been thought to reflect functional blocking of task-irrelevant pathways (Jensen and Mazaheri, 2010). However, Albu and Meagher’s (2016) findings may point toward an expectation-related inhibition of sensory processing or attention to somatic states, at least on a whole-brain level. Tu et al. (2019) also highlighted a role of alpha band activity, consistently with previous studies showing a clear link between sensory perception and alpha oscillations (Nir et al., 2010, 2012; Ai and Ro, 2014; Peng et al., 2015; Forschack et al., 2017). Moreover, Hird et al. (2018) found that SPN, an EEG correlate of imminent pain, was related to the nocebo responses, which points to a role of electrophysiological nociceptive processes under hyperalgesic conditions.

Biochemical correlates of nocebo hyperalgesia also reflect an involvement of primary sensory processing, although results appear less robust and generally have not been reproduced. Scott et al. (2008) demonstrated that nocebo hyperalgesia was characterized by a deactivation of the μ-opioid receptor system, in key nocebo-related brain areas such as the ACC, OFC, insula, amygdala, and PAG. This study further demonstrated that placebo analgesia was characterized by increased activations of the same systems in overlapping brain regions. However, these results should be interpreted with caution because the experimental paradigm did not purposely induce negative expectations, instead, findings are presented for those participants who showed nocebo responses upon a placebo manipulation. In an investigation of the contribution of biochemical correlates of nocebo hyperalgesia in the peripheral nervous system, Benedetti et al. (2014) found that nocebo hyperalgesia affected a specific biochemical pain pathway related to PG synthesis; however, in the absence of hypoxia-related activation of the COX-PG pathway, negative expectations were insufficient in initiating pain and PG synthesis. While these results highlighted a role of peripheral biochemicals that are directly related to pain signaling in nocebo hyperalgesia, they also pinpoint that nocebo hyperalgesia may be dependent on the intensity of an underlying pain.

Functional imaging studies have also implicated sensory discrimination in nocebo hyperalgesia, evident through the involvement of brain areas such as the thalamus and somatosensory cortex (Schmid et al., 2013, 2015; Egorova et al., 2015; Jensen et al., 2015). Interestingly, pain transmission via the spinal cord under nocebo hyperalgesic conditions also reveals vast similarities between the typical perception of a high pain stimulus and the perception of high pain resulting from expectations under hyperalgesic conditions (Geuter and Buchel, 2013; Tinnermann et al., 2017). Future studies could integrate the measures used in the abovementioned studies to cross-validate their results and achieve a more specific characterization of the various components involved in nocebo hyperalgesia. For example, peripheral components such as those found in Benedetti et al. (2014) may interact with peripheral or spinal components such as those discussed by Tinnermann et al. (2017) and a targeted manipulation of these variables could increase the robustness and interpretability of the current literature.

### Pain Integration and Modulation

While there is overlap between all pain processing components, a further possible categorization of neural mechanisms pertains to the central sensory modulation of pain. A consistent finding across the articles reviewed here is that nocebo hyperalgesia involves brain areas that are thought to be responsible for the modulation of pain signals (Lorenz et al., 2003; Ossipov, 2012; Jensen et al., 2015; Coulombe et al., 2017). Some of these key areas include the dlPFC, OFC, and PAG (Kong et al., 2008; Geuter and Buchel, 2013; Ellerbrock et al., 2015; Freeman et al., 2015). Egorova et al. (2015) discuss that the dlPFC involves down-stream circuits to, amongst others, the anterior insula, hypothalamus, and PAG, which are known to be involved in pain modulation (Atlas et al., 2010). Each of these areas has been implicated in nocebo hyperalgesia in the studies included in this review. Moreover, previous research does indicate a specific involvement of the rDLPFC in pain perception and cognitive evaluation of incoming stimuli (Lui et al., 2010; Kong et al., 2013). Importantly, in visceral pain studies, the involvement of the insula in nocebo hyperalgesia also marks mechanisms of sensory integration and cognitive pain evaluation (Craig, 2003; Wiech et al., 2010; Linnman et al., 2011). Thus, cognitive processing in frontal areas may interact with primary control centers for descending pain inhibition such as the PAG (Ossipov et al., 2014) thereby modulating pain under hyperalgesic conditions.

Findings involving the ACC deserve special attention, as they are most consistent across studies and indicate that higher order cognitive controls also play an important role in efferent pain modulation. The ACC is highlighted as a key region involved in cognitive pain processing (Keltner, 2006; Kong et al., 2008; Jensen et al., 2015; Tinnermann et al., 2017). Kong et al. (2008) showed that nocebo hyperalgesia was predominantly produced through a modulatory pain pathway involving the bilateral ACC. Based on the findings by Tinnermann et al. (2017) a modulatory function of the rACC on the descending pain system under hyperalgesic conditions is also evident. Notably, studies that include elaborate suggestions with a heavy negative load and hyperalgesic treatments, such as that of Kong et al. (2008), found an extensive involvement of cognitive pain processing, mediated through the ACC. Studies that did not employ extensive negative suggestions or sham treatments, however (Rodriguez-Raecke et al., 2010; Kong et al., 2013; Ellerbrock et al., 2015), showed a main involvement of pain-modulatory processes, but not affective processes, with a notable lack of ACC involvement. A possibility to replicate nocebo findings and evaluate the role of the ACC in hyperalgesia may exist, for example in patient studies or subdural electrode techniques that have been found to be powerful in locating sources of deep brain activity (Fahimi Hnazaee et al., 2020).

Concurrently, the spinal cord has also been found to interact with higher-order areas such as the PAG in nocebo hyperalgesia (Geuter and Buchel, 2013; Tinnermann et al., 2017). Pain modulation may thus involve an interconnected and wide-spread circuit, with nocebo studies showing both afferent pain amplification under nocebo conditions and efferent pain modulation (Geuter and Buchel, 2013; Tinnermann et al., 2017). This highlights a role of the entire pain system, from physiological nociceptive signaling in the spinal cord all the way to cognitive modulatory processing in the brain in nocebo hyperalgesia.

### Learning Leading to Expectations

Since the formation of negative expectations through learning lays at the core of nocebo hyperalgesia, it is unsurprising that cognitive modulation was found to be an important factor in nocebo hyperalgesia. MEG findings implicated alpha band connectivity between the rACC and MTG, which may reflect a process in which experience might be encoded through the dynamics of neural networks (Fries, 2005; Lange et al., 2012). Concurrently, analyses of EEG biomarkers (Thomaidou et al., 2021a) indicated a main involvement of long-range temporal correlations of brain oscillations as well as gamma band activity, both of which have previously been linked to learning (Miltner et al., 1999; Montez et al., 2009; Nikulin et al., 2012). These electrophysiological findings connect nocebo hyperalgesia to learning processes that can be reflected through electrophysiological components. Time-sensitive responses to nocebo hyperalgesia were studied through ERPs, which also highlighted the role of pain expectation. The reduction in N2/P2 amplitudes that was found by Pazzaglia et al. (2016), as well as the involvement of SPN found in Hird and colleagues, are linked to the predictability and temporal expectations of nociceptive stimuli (Wager et al., 2006; Iannetti et al., 2008), supporting the notion that nocebo effects are reliant on pain expectations. On the other hand, the finding of CNV differentiation between placebo and nocebo effects (Piedimonte et al., 2017) also highlighted the role of expectations, but additionally indicated that differential electrophysiological processes characterize learned expectation of analgesia and hyperalgesia. In sum, EEG and MEG studies provide support that learning is involved in nocebo hyperalgesia, while also highlighting the role of pain processing, at the electrophysiological level.

It is worth noting that the role of regions such as the amygdala and the hippocampus, that are perhaps best summarized in the context of (affective) learning, highlight the more refined and specific aspects of learning that underlie nocebo hyperalgesia. Many of the aforementioned relevant brain areas point toward integrative learning mechanisms being involved in nocebo hyperalgesia, including, for instance, the ACC and dlPFC. While it is generally accepted that learning plays a key role in nocebo hyperalgesia (Colloca et al., 2010; Evers et al., 2018; Blythe et al., 2019), unraveling the more exact learning correlates that contribute to the formation of nocebo effects is imperative. The amygdala and the hippocampus have specifically been implicated in aversive learning and conditions such as phobias, where fear learning plays a crucial role (Pissiota et al., 2003; Etkin and Wager, 2007; Onur et al., 2010). The role that the hippocampus plays in nocebo hyperalgesia (Kong et al., 2008; Jensen et al., 2015) relates to previous findings that the hippocampus mediates aversive learning (Goosens, 2011). This may in turn highlight an involvement of aversive learning processes in nocebo hyperalgesia. Moreover, within brain networks that include the ACC (Scott et al., 2008; Tinnermann et al., 2017), expectations and pain processing may be integrated in a way that involves the evaluation of sensory information based on learned negative expectations (Oliveira et al., 2007; Sallet et al., 2007; Onoda et al., 2008). In collaboration with the dlPFC, brain regions such as the ACC and the somatosensory cortex integrate information (Wiech et al., 2008) and are reportedly involved in expectation, anticipation, and error processing (Lorenz et al., 2003; Bingel et al., 2006), which are also essential elements of associative learning processes. At the same time, electrophysiological findings described in this review connect nocebo hyperalgesia to long-term learning processes (Albu and Meagher, 2016; Tu et al., 2019) as well as brain plasticity, with subcortical alpha-band oscillations engaging in rhythmic activities that have a plasticity function (Crunelli et al., 2018). These findings provide supporting physiological evidence of learning via association and long-term potentiation being central mediating factors of nocebo effects (Paulsen and Sejnowski, 2000; De Gennaro et al., 2008; Brincat and Miller, 2015).

### Anxiety and Fear

In the induction of nocebo hyperalgesia, anxiety and stress have long been thought to be modulatory factors. Benedetti et al. (2006), distinguished between different physiological anxiety pathways and pointed toward a potential distinction of HPA-mediated anxiety and anxiety related specifically to pain, with the latter being a potential contributor to nocebo hyperalgesia. However, the studies that investigated cortisol, a key chemical marker of stress states, found that, while cortisol seems to increase in response to pain or negative nocebo suggestions, there is no clear evidence in support of a modulatory role of the hormone on nocebo hyperalgesia (Benedetti et al., 2006, 2014).

The role of affective processing in nocebo hyperalgesia is marked by findings implicating the amygdala (Jensen et al., 2015; Schmid et al., 2015; Tinnermann et al., 2017), a primary region for fear processing and evaluation. While the amygdala has been implicated in nocebo effects by only a minority of studies, it is important to note that more threatening experimental contexts or verbal suggestions may potentially enhance the involvement of this brain region in nocebo effects. As such, it seems that fear, processed by amongst others the amygdala, may be a secondary modulatory factor in nocebo hyperalgesia. Moreover, the amygdala is extensively interconnected with areas that were consistently fount to be involved in nocebo hyperalgesia, such as the PFC, especially the OFC as well as the dlPFC (Salzman and Fusi, 2010). These areas may thus play an additive role in the processing of pain under nocebo hyperalgesic conditions, especially due to their involvement in cognitive-affective processes (Gray et al., 2002). The activation of the amygdala may not be essential, as frontal areas have also been shown to underlie nocebo hyperalgesia in the absence of an amygdala involvement (Freeman et al., 2015; Tinnermann et al., 2017). Nevertheless, findings have linked the activation of the amygdala to the magnitude of nocebo responses (Egorova et al., 2020) such that higher fear or anxiety seem to be linked to higher nocebo hyperalgesia. Future fMRI research could shed light on the role of fear and related physiological processes in the presentation of nocebo hyperalgesia by manipulating and directly comparing the threatening nature of the nocebo context.

### Limitations, Future Directions, and Clinical Implications

After summarizing the results reviewed here, it is important to note that the utilization of distinct learning methods for the induction of nocebo hyperalgesia may be influence the neurobiological findings of these experimental studies. In other fields of research, such as fundamental neuroscience in the domain of learning and memory, different types of learning have been shown to employ different brain processes and a complex architecture underlying distinct learning systems (Maestú et al., 2003; Schneider and Chein, 2003; Chein and Schneider, 2012; Clements-Stephens et al., 2012). Concurrently, differences in the affective load or valence of negative suggestions (Baeyens et al., 1992; Evers et al., 2018) or even potentially the magnitude of induced nocebo hyperalgesic effects (Petersen et al., 2014), may influence the physiological processes that are involved in the induction and evocation of nocebo responses. Few studies have systematically studied these methodological aspects of nocebo effects. The knowledge base on nocebo hyperalgesia could significantly benefit from future research focusing on replication, comparability between studies, and an examination of the influence that methodological aspects have on the neurobiological nocebo correlates. While the field of nocebo hyperalgesia is young, it is therefore also a contemporary field of science that could benefit by setting an example in replicating findings and compiling consistent and reliable results.

Overall, one important future aim for nocebo studies may involve the systematic examination of learning, as this seems to be a major factor underlying nocebo hyperalgesia. This implication of learning is important, particularly in light of evidence-based theories that show how the social environment and interpersonal experiences shape the experience of pain in healthy and patient populations (Karos et al., 2015; Karos, 2017; Vervoort et al., 2018). The specific learning correlates that are involved in nocebo effects have not been systematically manipulated and studied. Pharmacological and cognitive manipulations of learning are effective means in which learning has been studied in other fields of research (Davis, 2006; Norberg et al., 2008). Yet, several important biochemicals relevant for pain and/or cognitive processes have not yet been studied. What future nocebo studies could attempt, is a direct manipulation of learning via, for example, agents that affect the n-methyl-D-aspartate receptor system such as amino acids (Brom et al., 2015). Alternatively, direct measures of learning ability (such as the Weschler Memory Scale; Wechsler, 2014) may shed light onto the specific learning mechanisms that are involved, and how individual differences related to learning may facilitate the formation of nocebo effects. It is imperative for future research to focus on precise learning mechanisms and comparisons between learning mechanisms in order to better understand and potentially therapeutically target the fundamental mechanisms by which nocebo hyperalgesia is induced.

While some of the neurobiological correlates of nocebo effects are beginning to unravel, application of this knowledge in clinical contexts is also receiving increased attention. The studies reviewed in this article provide important insights into the key neurobiological mechanisms involved in nocebo effects. Notably, some of the neural correlates that have been linked to nocebo hyperalgesia are also implicated in chronic pain. For instance, data suggest that chronic pain involves brain structures such as the cingulate cortex and hippocampus may be associated with chronic stress and mesolimbic dopamine abnormalities that are involved in processing both nociceptive signaling and affective components of pain (Wood, 2004; Gondo et al., 2012; Schmid et al., 2015; Sturgeon et al., 2016; Vogt, 2016). Such overlapping neural factors may be crucial in the search for biomarkers of nocebo effects and identifying reliable risk factors for its formation. Considering the mechanisms of action of nocebo hyperalgesia may significantly aid the process of preventing or counteracting nocebo effects in pain patients.

## Conclusion

We conducted a comprehensive review of the currently known neurobiological correlates of nocebo hyperalgesia. Functional studies showed that pain-related activity might be amplified already in the spinal cord and further modulated by higher cognitive representations. Electrophysiological findings, though limited, also pointed toward involvement of cognitive-affective processes. Neurochemical findings were not consistent on whether cortisol may play a role in nocebo hyperalgesia. These findings are an important step in identifying the neurobiological mechanisms through which nocebo effects may exacerbate pain. Nevertheless, one major limitation arising from the compilation of neurobiological findings from the nocebo literature is the inconsistency in methods and results. Future studies in this field should consider not only the pressing need for consistency and reproduction of findings, but also the need for transparency about what findings reflect. Traceable and consistent methods and results in neurobiological nocebo studies are necessary in order for a reliable picture to be drawn. A better understanding of nocebo effects on pain might eventually lead to the development of methods to identify, minimize or prevent nocebo effects on pain.

## Author Contributions

MT: conceptualization, data collection and extraction, methodology, visualization, writing, and review and editing. KP: conceptualization, supervision, methodology, writing, and review and editing. MK: data collection and extraction, methodology, writing, and review and editing. AE: conceptualization, supervision, funding acquisition, methodology, and review and editing. DV: conceptualization, supervision, methodology, writing, and review and editing. All authors contributed to the article and approved the submitted version.

## Conflict of Interest

The authors declare that the research was conducted in the absence of any commercial or financial relationships that could be construed as a potential conflict of interest.

## Supplementary Material

Supplementary Table 1 Overview of study characteristics included in the review and key findings. These selected studies examined experimentally induced nocebo hyperalgesia in healthy humans. The studies are categorized as described in the main text, based on neurobiological outcome and subsequently based on their relevance to each other. In all studies, nocebo effects were evoked and assessed during a phase where all pain stimulations were equivalent intensity. The number of trials and length of pain stimulations at plateau are also listed. All studies employed diverse nocebo induction paradigms and conditioning paradigms employed a range of different numbers of stimuli and pain application methods. ^∗^The column listing nocebo acquisition trials also lists acquisition (i.e., reinforced) trials included within evocation phases when applicable. Abbreviations: NVS, Negative (Verbal) Suggestions; EEG, electroencephalography; MEG, magnetoencephalography; PET, Positron Emission Tomography; tDCS, transcranial Direct Current Stimulation; fMRI, functional Magnetic Resonance Imaging.
