# Supplementary material for: How Negative Experience Influences the Brain: A Comprehensive Review of the Neurobiological Underpinnings of Nocebo Hyperalgesia
Source: Front Neurosci. 2021 Mar 24;15:652552. doi: 10.3389/fnins.2021.652552 (PMC8024470; doi:10.3389/fnins.2021.652552)
Supplement: Supplementary Table 1 — Overview of study characteristics included in the review and key findings. These selected studies examined experimentally induced nocebo hyperalgesia in healthy humans. The studies are categorized as described in the main text, based on neurobiological outcome and subsequently based on their relevance to each other. In all studies, nocebo effects were evoked and assessed during a phase where all pain stimulations were equivalent intensity. The number of trials and length of pain stimulations at plateau are also listed. All studies employed diverse nocebo induction paradigms and conditioning paradigms employed a range of different numbers of stimuli and pain application methods. ∗The column listing nocebo acquisition trials also lists acquisition (i.e., reinforced) trials included within evocation phases when applicable. Abbreviations: NVS, Negative (Verbal) Suggestions; EEG, electroencephalography; MEG, magnetoencephalography; PET, Positron Emission Tomography; tDCS, transcranial Direct Current Stimulation; fMRI, functional Magnetic Resonance Imaging. [file Table_1.docx]

**SUPPLEMENTARY TABLE**

| First author | Year | Sample size per nocebo group (n) | Experimental paradigm per nocebo group | Pain administration  method | Nocebo (high pain) acquisition trials  (conditioning paradigms) * | Neuro-physiological measure | Key neurobiological outcome measures | Key findings |
| --- | --- | --- | --- | --- | --- | --- | --- | --- |
| Albu | 2016 | 15 | NVS | Thermal | n/a | EEG | Resting-state EEG with classical frequency band analyses | Enhanced low alpha  (8–10 Hz) power |
| Thomaidou, Blythe,  Houtman | 2021 | 36 | Conditioning with NVS | Thermal | 16 trials  (10-second plateau) | EEG | Continuous and resting-state EEG with classical frequency band and biomarker analyses | Increases in complexity of oscillations leading to larger nocebo responses, increases in alpha oscillations in nocebo-augmented pain |
| Pazzaglia | 2016 | 9 & 9 | NVS & Conditioning with NVS | Laser | 50-60 trials  (<1-second plateau) | EEG | Laser-evoked potentials that are responses to laser radiant heat pulses and reflect activation of Aδ nociceptors | N2/P2 amplitude reduction |
| Piedimonte | 2017 | 17 & 17 | NVS & Conditioning with NVS | Electric | 20 trials in conditioning  4 trials in evocation  (<1-second plateau) | EEG | Early and late contingent negative variation amplitudes that relate to sensory and motor components of pain, respectively | Contingent negative variation amplitudes showed higher early negativity in nocebo trials |
| Hird | 2018 | 14 | Conditioning | Laser & Electric | 90 trials in conditioning/evocation  (<1-second plateau) | EEG | Laser-evoked potentials and electric evoked potentials (as above), specifically the stimulus-preceding negativity component, related to processing of imminent pain. | Stimulus-preceding negativity at centroparietal electrodes was found to differentiate pain intensity expectation, with nocebo trials linked to lower amplitudes |
| Tu | 2019 | 21 | Conditioning & Observational learning | Thermal | 20 trials in  direct conditioning  (2-second plateau) | MEG | Resting-state MEG, pre- and post- the nocebo manipulation | Decreased alpha connectivity between the left rostral anterior cingulate cortex and left middle temporal gyrus |
| Benedetti | 2006 | 37 | NVS | Ischemic | n/a | Pharmacology | Pharmacological manipulations and measurement of adrenocorticotropic hormone and cortisol plasma concentrations | Increased adrenocorticotropic hormone and cortisol plasma concentrations |
| Benedetti | 2014 | 35 | Socially induced NVS | Hypoxia-induced headache | n/a | Biochemical | Pharmacological manipulations and measurement of Salivary PG, TXA2, and cortisol | Prostaglandin, thromboxane A_2_, and salivary cortisol increases |
| Scott | 2008 | 5 | NVS | Needle punctures | n/a | PET | Measurement of μ-opioid and dopamine (D2/D3 receptor) neurotransmission | Deactivation of μ-opioid and mesolimbic dopamine neurotransmission |
| Egorova | 2015 | 30 | Conditioning & Nonconscious evocation | Thermal | 22 trials in conditioning  (4-second plateau) | tDCS | Manipulation of brain activity via electrical currents that increase and decrease neuronal excitability in the DLPFC | Involvement of dorsolateral prefrontal cortex activation |
| Keltner | 2006 | 13 | Conditioning | Thermal | 5 trials in preconditioning  10 trials in conditioning  (30-second plateau) | fMRI | Differences in BOLD activations | Increased activation in caudal anterior cingulate cortex and cerebellum, and enhanced activation of afferent pain circuitry at the thalamic level |
| Kong | 2008 | 13 | Conditioning with NVS | Thermal | 6 trials in conditioning  2 trials in evocation  (7-second plateau) | fMRI | Differences in BOLD activations and connectivity analyses | Decreased activations in dorsolateral prefrontal cortex and orbitofrontal cortex. Increased activations in mid temporal gyrus, insula, anterior cingulate, hippocampus |
| Kong | 2013 | 46 | Conditioning | Thermal | 4 trials in conditioning  3 trials in evocation  (12-second plateau) | fMRI | Differences in BOLD activations | Functional connectivity between frontoparietal regions and rostral anterior cingulate /medial prefrontal cortex was associated with nocebo |
| Rodriguez-Raecke | 2010 | 38 | NVS | Thermal | n/a | fMRI | Differences in BOLD activations | Increased activation of the operculum. |
| Ellerbrock | 2015 | 20 | NVS | Thermal | n/a | fMRI | Differences in BOLD activations and connectivity analyses | Increased activation of the operculum. Deactivation of the periaqueductal grey. |
| Freeman | 2015 | 24 | Conditioning with NVS | Thermal | 3 trials in conditioning  1 trial in evocation  (7-second plateau) | fMRI | Differences in BOLD activations | Increased activations in insula, orbitofrontal cortex, and periaqueductal grey |
| Jensen | 2015 | 24 | Conditioning & Nonconscious evocation | Thermal | 25 trials in conditioning  (4-second plateau) | fMRI | Differences in BOLD activations | Increased activations in insula, anterior cingulate cortex, thalamus, brainstem, amygdala, and hippocampus |
| Egorova | 2020 |  | Conditioning with NVS | Thermal | 3 trials in conditioning  1 trial in evocation  (7-second plateau) | fMRI | Differences in BOLD activations and connectivity analyses | Increased amygdala- striatum connectivity correlated with magnitude of nocebo responses. |
| Schmid | 2013 | 18 | NVS | Visceral pressure | n/a | fMRI | Differences in BOLD activations | Increased insula and somatosensory cortex activation |
| Schmid | 2015 | 22 | NVS | Visceral pressure | n/a | fMRI | Differences in BOLD activations and connectivity analyses | Hyperactivation in somatosensory cortex, dorsolateral prefrontal cortex, midcingulate cortex, posterior cingulate cortex, insula, thalamus, and amygdala |
| Geuter | 2013 | 23 | Conditioning with NVS | Thermal | 6 trials in preconditioning  6 trials in conditioning  (17-second plateau) | Spinal fMRI | Differences in spinal BOLD activations | Increased activation in the ipsilateral dorsal horn of the spinal cord. |
| Tinnermann | 2017 | 49 | Conditioning with NVS | Thermal | 16 trials in preconditioning  16 trials in conditioning  (16-second plateau) | Spinal and brain fMRI | Differences in brain and spinal BOLD activations | Increased activation in spinal cord, slightly more caudal and medial than pain cluster. Increased activation in prefrontal areas, amygdala, periaqueductal gray. Deactivation of the rostral anterior cingulate cortex. |
