# Supplementary material for: How Negative Experience Influences the Brain: A Comprehensive Review of the Neurobiological Underpinnings of Nocebo Hyperalgesia
Source: Front Neurosci. 2021 Mar 24;15:652552. doi: 10.3389/fnins.2021.652552 (PMC8024470; doi:10.3389/fnins.2021.652552)
Supplement: Supplementary file 2 [file Data_Sheet_1.DOCX]

Supplementary Material

**1 Search terms**

1.1 PubMed search

("Nocebo Effect"[Mesh] OR Nocebo[Title/Abstract] OR Expectation[Title/Abstract] OR Expectations [Title/Abstract] OR Expectancies[Title/Abstract] OR Expectations[Title/Abstract] OR "Conditioning (Psychology)"[Mesh:noexp] OR "Conditioning, Classical"[Mesh] OR (conditioning[tiab] NOT medline[sb]) OR "Verbal Suggestion"[Title/Abstract] OR "Verbal Suggestions"[Title/Abstract]) OR "Negative Suggestion"[Title/Abstract] OR " Negative Suggestions"[Title/Abstract]) AND ("Pain"[Mesh] OR Pain[Title/Abstract] OR Nocicept*[Title/Abstract] OR Hyperalges*[Title/Abstract]) AND (dopamine*[Title/Abstract] OR seroton*[Title/Abstract] OR 5HT[Title/Abstract] OR cholecystokinin[Title/Abstract] OR cck[Title/Abstract] OR opioid[Title/Abstract] OR µ-opioid[Title/Abstract] OR hypothalamic[Title/Abstract] OR hpa[Title/Abstract] OR cortisol[Title/Abstract] OR adrenocorticotropic[Title/Abstract] OR epinephrine[Title/Abstract] OR noradrenaline[Title/Abstract] OR noradrenergic[Title/Abstract] OR pharmacolog*[Title/Abstract] OR neuropharmacolog*[Title/Abstract] OR neurochemic*[Title/Abstract] OR chemical[Title/Abstract] OR neurochemicals[Title/Abstract] OR chemistry[Title/Abstract] OR neurochemistry[Title/Abstract] OR neurotransmitter[Title/Abstract] OR neurotransmitters[Title/Abstract] OR neurobiology[Title/Abstract] OR neurobiological[Title/Abstract] OR endocrine[Title/Abstract] OR hormone*[Title/Abstract] OR hormonal[Title/Abstract] OR "neural mechanisms"[Title/Abstract] OR oxytocin[Title/Abstract] OR testosterone[Title/Abstract] OR immune[Title/Abstract] OR immunity[Title/Abstract] OR autoimmune[Title/Abstract] OR autoimmunity[Title/Abstract] OR PET[Title/Abstract] OR fMRI[Title/Abstract] OR SPECT[Title/Abstract] OR resonance[Title/Abstract] OR tomography[Title/Abstract] OR EEG[Title/Abstract] OR electroencephalography[Title/Abstract] OR DTI[Title/Abstract] OR transcranial[Title/Abstract] OR hippocampus[Title/Abstract] OR hypothalamus[Title/Abstract] OR "cingulate cortex"[Title/Abstract] OR spinal[Title/Abstract])

1.2 PsychInfo search

(nocebo/ OR classical conditioning/ OR (nocebo effect OR nocebo effects OR nocebo response OR nocebo responses OR nocebo hyperalgesia OR expectations OR expectancies OR classical conditioning OR verbal suggestion OR verbal suggestions).ti,ab.) AND (pain/ OR hyperalgesia/ OR (pain OR hyperalgesia OR hyperalgesic).ti,ab.) AND (dopamine OR serotonin OR 5HT OR cholecystokinin OR cck OR opioid OR µ-opioid OR hypothalamic OR hpa OR cortisol OR adrenocorticotropic OR epinephrine OR noradrenaline OR noradrenergic OR pharmacological OR neuropharmacological OR neurochemical OR chemical OR neurochemicals OR chemistry OR neurochemistry OR neurotransmitter OR neurotransmitters OR neurobiology OR neurobiological OR endocrine OR hormone* OR hormonal OR "neural mechanisms" OR oxytocin OR testosterone OR immune OR immunity OR autoimmune OR autoimmunity OR PET OR fMRI OR SPECT OR resonance OR tomography OR neuroimaging OR EEG OR electroencephalography OR DTI OR transcranial OR hippocampus OR hypothalamus OR "cingulate cortex" OR spinal .ti,ab)
